# Prognostic biomarkers in squamous cell carcinoma of the anus: a systematic review

**DOI:** 10.1038/sj.bjc.6605984

**Published:** 2010-11-09

**Authors:** T Lampejo, D Kavanagh, J Clark, R Goldin, M Osborn, P Ziprin, S Cleator

**Affiliations:** 1 Department of Biosurgery and Surgical Technology, Imperial College London, St Mary's Hospital, Praed Street, London, W2 1NY, UK; 2 Department of Pathology, Imperial College London, St Mary's Hospital, Praed Street, London, W2 1NY, UK; 3 Department of Oncology, Imperial College London, St Mary's Hospital, Praed Street, London, W2 1NY, UK

**Keywords:** prognosis, biomarker, molecular marker, anal carcinoma

## Abstract

**Background:**

Recent decades have seen combination chemoradiotherapy become the standard treatment for anal squamous cell carcinoma (SCC). However, the burden of this disease continues to rise, with only 10% of patients with metastatic disease surviving >2 years. Further insight into tumour characteristics and molecular biology may identify novel therapeutic targets. This systematic review examines current prognostic markers in SCC of the anus.

**Methods:**

An extensive literature search was performed to identify studies reporting on biomarkers in anal cancer in the context of clinical outcome following treatment primarily with chemoradiotherapy.

**Results:**

In all, 21 studies were included. A total of 29 biomarkers were studied belonging to 9 different functional classes. Of these biomarkers, 13 were found to have an association with outcome in at least one study. The tumour-suppressor genes *p53* and *p21* were the only markers shown to be of prognostic value in more than one study.

**Conclusions:**

An array of biomarkers have been identified that correlate with survival following chemoradiotherapy in anal cancer. However, investigators are yet to identify a biomarker that has the ability to consistently predict outcome in this disease. Further studies are needed to elucidate whether these candidate biomarkers demonstrate their optimum value when they serve as targets for new therapeutic strategies.

Anal cancer is a disease whose incidence has risen markedly in several parts of the world including Europe and the United States (Rousseau *et al*, 2005). Of notable concern is the significant impact of this disease on the young male population, with incidence rates of up to 37 per 100 000 in homosexual men (Place *et al*, 2001). Of men who have sex with men (MSM), 95% are seropositive for the implicated viral pathogen, human papilloma virus (subtypes 16, 18, 32 and 34) (Palefsky *et al*, 2005). Also, 81% of MSM have anal intraepithelial neoplasia (AIN; premalignant lesion) (Palefsky *et al*, 2005). HPV is associated with the proteins E6 and E7 that can silence important tumour-suppressor proteins in normal cells (Mammas *et al*, 2008). These proteins, namely p53 and Rb, normally exert apoptotic cellular effects to prevent disordered cell growth (Mammas *et al*, 2008). Removal of this growth restriction may lead to malignant transformation (Mammas *et al*, 2008). At the time of diagnosis, up to 50% of patients have locoregional disease with visceral metastases present in 10% of patients (UKCCCR Anal Cancer Trial Working Party, 1996; Rousseau *et al*, 2005). The most common sites are the liver and lungs (Rousseau *et al*, 2005).

In the past, the mainstay of treatment for anal carcinoma was abdominoperineal resection with formation of a permanent end colostomy. Over the last two decades, combined chemoradiotherapy pioneered in the 1970s by Nigro *et al* (1974) has become the standard of care with salvage abdominoperineal resection reserved for a small proportion of cases refractory to chemoradiotherapy (Place *et al*, 2001). Despite respectable treatment response rates in non-metastatic disease with overall survival rates of 60–75%, only 10% of patients with distant metastases survive 2 years from the time of their diagnosis (Cummings, 2006; Das *et al*, 2008). Furthermore, it is evident that treatment response shows a degree of heterogeneity between individual patients within a specific stage category, suggesting a spectrum of chemoradiation sensitivity. Furthermore, the acute and long-term toxicity associated with standard chemoradiation schedules are considerable, and therefore predictors of response and novel therapeutic approaches are required to allow treatments to be tailored according to treatment sensitivity.

Clinical and pathological factors have been evaluated extensively with regard to outcomes in anal cancer. Clinicopathological parameters have not consistently enabled prediction of response to currently available treatment modalities. This had led to considerable interest in prognostic and predictive biomarkers as putative means to improving patient survival. A prognostic biomarker gives information regarding the patient's overall outcome, irrespective of the therapy received, whereas a predictive biomarker provides information about the effect of a specific therapeutic intervention (Oldenhuis *et al*, 2008). Molecular analysis may permit the tailoring of treatments to individual patients and help identify new therapeutic targets. Tyrosine kinases (TKs), which have an established role in malignant transformation of human cells, have served as major target in cancer treatment strategies such as imatinib, the BCR-ABL TK inhibitor used in the treatment of chronic myeloid leukaemia and GISTs (Madhusudan and Ganesan, 2004). In recent years, the epidermal growth factor receptor (EGFR – particularly HER-1 and HER-2) has been the most extensively investigated TKs and now forms a significant component of the ongoing research into molecular targeted cancer therapy. In non-small cell lung cancer, mutations in the TK domain of the EGFR have been shown to be associated with a high treatment response rate to the EGFR TK inhibitor erlotinib (Paz-Ares *et al*, 2006). Randomised trials in metastatic colorectal cancer have demonstrated that use of monoclonal antibodies directed against EGFR (HER-1), namely cetuximab and panitumumab, is associated with favourable outcomes in patients expressing the wild-type form of K-ras proto-oncogene (Siddiqui and Piperdi, 2009). Therefore, a search for putative predictive markers in anal cancer is warranted in order to explore whether these and other targeted treatments should be assessed in the setting of early and/or metastatic anal cancer.

The purpose of this systematic review was to evaluate the literature available to-date on biological and molecular prognostic factors in squamous cell carcinoma (SCC) of the anus.

## Materials and methods

A systematic review was undertaken to identify the prognostic significance of biomarkers in anal cancer. During this process, reference to the AMSTAR measurement tool was made (Shea *et al*, 2007). This 11-component assessment tool was constructed to assess the methodological quality of systematic reviews. Although the reproducibility and construct validity of this tool is currently uncertain, preliminary findings suggest that AMSTAR has good construct validity and is a reliable tool for the methodological assessment of systematic reviews.

A comprehensive literature search was performed by two independent reviewers (TL and DK) using the PubMed, Web of Science, Embase and Google Scholar databases. This search was conducted up to 9 January 2010. The following keywords were used in various combinations: ‘anal cancer’, ‘biomarkers’, ‘molecular markers’, ‘biological markers’, ‘prognosis’ and ‘clinical outcome’ in addition to searching for individual biomarkers. Further relevant studies were identified by searching the references cited in identified publications and also by utilisation of the PubMed ‘related articles’ tool (Shalhoub *et al*, 2009).

No exclusions from the analysis were made based on the date of the publication or the type of the study. Articles not published in the English language were excluded. Studies were excluded because of the absence of (1) any data regarding clinical outcome or prognosis; (2) potentially extractable or a complete set of data considered necessary; (3) peer review status; and (4) the use of chemotherapy/radiotherapy as a primary treatment. Studies were also excluded if they referred to AIN rather than carcinoma.

In addition to patient demographics and tumour characteristics, outcomes of interest related to the study of potential biomarkers included the type of treatment received, tumour recurrence and patient relapse rates, disease-free survival (DFS), overall survival and mortality.

## Results

**Figure 1:**
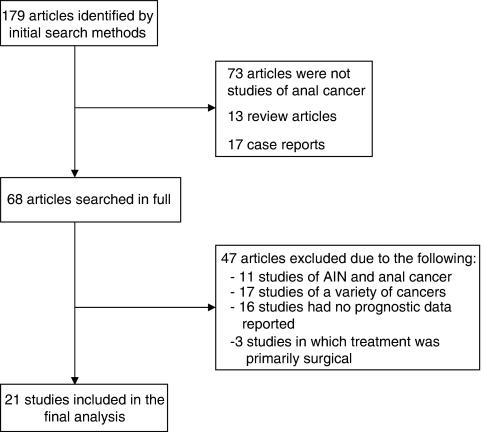
Flow diagram representing the selection process for the studies included in the final analysis.

Using the search methods described above, a total of 179 articles were identified. Of these, 73 were initially excluded as they were not studies of anal cancer. Eight articles were not related to the study of biomarkers. A further 30 articles were also excluded by abstract review; 13 of these were review articles and 17 were case reports. Of the remaining 68 references searched in full, 11 studies were excluded as they included the premalignant form, AIN, with no discrimination of results between the two disease groups. Another 17 studies were excluded as they combined data from human cancers of different anatomical locations (including anal carcinoma) with no breakdown of results for the various cancer types. Sixteen studies of biomarkers in anal cancer were excluded as correlation with prognosis was not assessed. Finally, three studies failed to meet the inclusion criteria, as the primary form of treatment in these studies was radical surgery rather than chemoradiation. Ultimately, 21 studies remained for full inclusion in this literature review (Figure 1) (Fontana *et al*, 1991; Tanum *et al*, 1992; Goldman *et al*, 1993; Holm and Tanum, 1996; Tanum and Holm, 1996; Allal *et al*, 1998, 2003, 2004; Grabenbauer *et al*, 1998; Bonin *et al*, 1999; Indinnimeo *et al*, 1999, 2000a, 2000b, 2001; Wong *et al*, 1999; Holm *et al*, 2001; Le *et al*, 2005; Alvarez *et al*, 2006; Nilsson *et al*, 2006; Bruland *et al*, 2008; Ajani *et al*, 2009).

**Figure 2:**
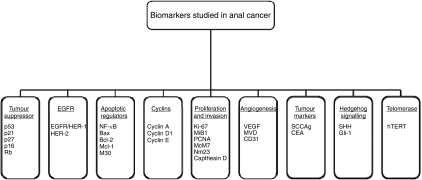
The classes and subclasses of biomarkers evaluated in the final 21 studies (highlighted in bold are the biomarkers that were associated with outcome in at least one study).

The 21 studies included in the analysis evaluated a number of different of biomarkers, which are discussed below and illustrated in Figure 2.

### Tumour-suppressor genes

The primary role of tumour-suppressor genes is to regulate cell division, differentiation and apoptosis in order to prevent genetic damage that may lead to the development of cancerous cells. *p53*, *p21*, *p27*, *p16* and the *retinoblastoma* (*RB*) gene are examples of such genes whose prognostic significance has been studied in anal carcinoma.

#### p53

The *p53* gene located on chromosome 17p13.1 encodes a protein that has an important role in cell cycle regulation and apoptosis (Kastan *et al*, 1995). Additionally, wild-type p53 has been linked with cellular DNA damage repair and inhibition of angiogenesis (Vogelstein *et al*, 2000). This gene has been extensively studied in several human cancers, and mutations of this gene are considered to be the commonest genetic anomaly in human tumours (Wong *et al*, 1999). Evidence suggests that mutated p53 can lead to genomic instability and permit malignant transformation of previously non-cancerous cells (Finlay *et al*, 1989).

Eight studies evaluated the prognostic significance of p53 in anal carcinoma (Table 1) (Tanum and Holm, 1996; Bonin *et al*, 1999; Indinnimeo *et al*, 1999; Wong *et al*, 1999; Allal *et al*, 2003; Le *et al*, 2005; Nilsson *et al*, 2006; Ajani *et al*, 2009). Immunohistochemistry was utilised in all studies to assess nuclear p53 status in anal carcinoma paraffin-embedded specimens. Wild-type p53 protein has a relatively short half-life and is less readily detectable by immunohistochemical staining. However, mutated p53 typically undergoes a conformational change that stabilises the protein, resulting in nuclear accumulation and therefore positive staining (Neal *et al*, 2006). In these studies, p53 was considered to be overexpressed if >5% of tumour cells stained positive for nuclear p53. The proportion of patients with anal carcinoma overexpressing p53 ranged from 34 to 100%.

Two studies demonstrated a significant correlation between p53 status and clinical outcome in anal carcinoma. Allal *et al* (2003) found through multivariate analysis that patients with p53-positive tumours had a lower rate of locoregional control (RR, 0.38; *P*=0.03) and shorter DFS (RR, 0.29; *P*=0.003). Similarly, Wong *et al* (1999) showed that p53 overexpression was an independent adverse marker for DFS in patients with anal carcinoma treated with combined chemoradiotherapy (*P*=0.01). Another study consisting of 64 patients reported a trend using univariate analysis towards higher locoregional failure rates following chemoradiotherapy in patients with mutant p53 compared with those with the wild-type form (48 *vs* 27%, *P*=0.14) (Bonin *et al*, 1999). However, this finding did not reach statistical significance and no further correlation was seen with either DFS or overall survival (Bonin *et al*, 1999). The five remaining studies examining p53 did not identify any correlation between its overexpression and clinicopathological parameters or survival (Tanum and Holm, 1996; Indinnimeo *et al*, 1999; Le *et al*, 2005; Nilsson *et al*, 2006; Ajani *et al*, 2009).

#### p21

Activation of the p21 protein, a cyclin-dependent kinase (CDK) inhibitor, results in cell cycle arrest at the G1- to S-phase transition in mammals (Harper *et al*, 1993). Its effector functions are predominantly induced by p53 and it is considered to be a mediator of the tumour-suppressor activity of p53 (el-Deiry *et al*, 1993). However, it also appears to function via mechanisms independent of the *p53* gene in response to cellular DNA damage (Roninson, 2002). Underexpression of p21 has been associated with poor prognostic outcome in various human cancers including SCC of the lung (Komiya *et al*, 1997).

Three studies have assessed the relationship between p21 and prognosis in anal carcinoma using immunohistochemistry to determine the level of p21 expression (Table 2) (Holm *et al*, 2001; Allal *et al*, 2004; Nilsson *et al*, 2006). The study by Holm *et al* (2001) included 94 patients with anal carcinoma. They reported high p21 expression in 71% of patients with anal carcinoma (*vs* 10% positivity in negative controls of normal anal squamous epithelium). Univariate analysis demonstrated a significant association between lack of p21 immunoreactivity and poorly differentiated carcinomas (*P*=0.004) and shorter overall survival (*P*=0.013) (Holm *et al*, 2001). No significant correlation was seen between p21 expression and tumour stage or presence of nodal metastases in this study (Holm *et al*, 2001). Nilsson *et al* (2006) and Allal *et al* (2004) reported high p21 expression in 69 and 65% of anal cancers, respectively. Both studies demonstrated a trend towards shorter survival in p21-negative tumours, but statistical significance was not reached (*P*=0.08 and *P*=0.1, respectively). Conversely, multivariate analysis by Nilsson *et al* (2006) found that patients with tumours underexpressing p21 had a significantly higher locoregional failure rate (*P*<0.05).

#### p27

The p27 protein is a member of the CDK inhibitor family that blocks phosphorylation of the Rb protein, thus inhibiting cell growth and proliferation (Coate *et al*, 2009). Like the p21 protein, p27 is involved in cell cycle control at the G1- to S-phase transition (Coate *et al*, 2009). A Norwegian study assessed the prognostic significance of p27 using immunohistochemistry in 94 patients with anal carcinoma (Table 2) (Holm *et al*, 2001). Nuclear p27 staining was detected in 75% of cases of anal carcinoma compared with 100% of samples of normal anal squamous epithelium (Holm *et al*, 2001). No correlation was found between p27 expression and tumour stage, lymph node involvement or overall survival (Holm *et al*, 2001). The investigators did however find that reduced p27 expression was associated with moderate/highly differentiated carcinomas (Holm *et al*, 2001).

#### p16

The p16 protein, also known as cyclin-dependent kinase inhibitor 2A (CDKN2A), specifically targets and inhibits the activity of CDK 4 and CDK 6, both of which are involved in cell proliferation (Bruland *et al*, 2008). Studies of p16 have demonstrated a potential prognostic role for this biomarker in oropharyngeal SCC (Shi *et al*, 2009). Two recent studies consisting of 55 and 30 patients, respectively, investigated the possible role of p53 in predicting outcome in anal cancer (Table 2) (Bruland *et al*, 2008; Ajani *et al*, 2009). Neither study identified any association between p16 protein expression and clinical outcome or treatment response (Bruland *et al*, 2008; Ajani *et al*, 2009).

#### The *RB* gene

The *RB* gene, a tumour-suppressor gene originally found to be mutated in the rare paediatric malignancy, retinoblastoma, encodes a nuclear protein involved in cell differentiation, proliferation and apoptosis (Du and Searle, 2009). *RB* gene mutations leading to loss of its tumour-suppressor function has been identified in a variety of human malignancies (Bourgo *et al*, 2009; Du and Searle, 2009). One study of the *RB* gene in anal carcinomas found no significant rearrangement or loss of the RB locus in malignant (or benign) tissue (Crook *et al*, 1991).

Tanum and Holm (1996) evaluated 97 patients treated with chemoradiotherapy for anal carcinoma (Table 2). Of the pre-treatment tumour specimens, 95% stained positive for the Rb protein. No relationship was identified between protein expression and the clinical course of the disease.

### EGFR

Four transmembrane TK proteins (HER-1 to HER-4) make up the EGFR family of receptors that regulate cellular proliferation, angiogenesis, apoptosis and migration via binding of specific ligands (Neal *et al*, 2006; Coate *et al*, 2009). The intimate association between the EGFR overexpression and cancer development has served as a platform for the introduction of newer therapies targeting these receptors. This is therapeutically achieved through direct inhibition of TK or through antibody-mediated targeting of receptors. Monoclonal antibodies directly bind to the external domain of these TK receptors. Traztuzumab (Herceptin), a monoclonal antibody against the HER-2 receptor, has been widely established in breast cancer patients overexpressing HER-2 with significantly improved survival rates reported in these patients (Treish *et al*, 2000). As previously mentioned, the TK inhibitor erlotinib has been successfully used in the treatment of non-small cell lung cancer (Paz-Ares *et al*, 2006).

Tanum and Holm (1996) studied HER-2 protein expression in 97 patients with anal carcinoma who received chemoradiotherapy. HER-2 protein expression was undetectable (Table 3) (Tanum and Holm, 1996). Similarly, in a Canadian study of 21 patients with anal cancer, all samples were negative for HER-2 (Le *et al*, 2005). However, this study found that EGFR (HER-1) was strongly expressed in 100% of patients (Le *et al*, 2005). Ajani *et al* (2009) also performed immunohistochemical analysis of EGFR and reported expression in 86% of patients with anal cancer, but no significant correlation was observed between the degree of EGFR staining and DFS. One study detected EGFR using immunohistochemistry in 55% of anal SCCs. They also performed fluorescent *in situ* hybridisation (FISH) to assess EGFR gene copy numbers (Alvarez *et al*, 2006). There was no correlation between EGFR status and clinicopathological parameters. Furthermore, there was a lack of concordance between the two detection techniques (Alvarez *et al*, 2006).

### Regulators of apoptosis

Apoptosis and the genes that regulate it exert a profound effect on the malignant phenotype in mammalian cells (Lowe and Lin, 2000) (Table 4). Mutations in genes encoding these apoptotic regulatory proteins can lead to tumour initiation, progression or metastasis (Lowe and Lin, 2000).

#### Nuclear factor-*κ*B (NF-*κ*B)

The nuclear transcription factor NF-*κ*B has been shown to prevent apoptosis through direct inhibitory mechanisms as well as indirectly by blocking mitochondria-mediated apoptosis through neutralisation of reactive oxygen species (Naugler and Karin, 2008). Moreover, poor chemotherapeutic responses have been attributed to NF-*κ*B-induced resistance to apoptosis (Naugler and Karin, 2008).

A recent study (Ajani *et al*, 2009) of 30 patients with anal cancer treated with chemoradiotherapy reported that NF-*κ*B was an independent predictor of DFS. Multivariate analysis revealed that patients with higher NF-*κ*B levels, detected by immunohistochemistry, had shorter DFS (*P*=0.002) (Ajani *et al*, 2009).

#### Bcl-2, Bax and M30

The Bcl-2 family consists of at least 15 protein members including Bcl-2 and Mcl-1 that promote cell survival by inhibiting apoptosis. They are upregulated in certain tumour types (Lowe and Lin, 2000). Conversely, the Bax protein is a proapoptotic molecule that functions as a downstream component of the p53 pathway. It has been shown to be mutationally inactivated in patients with SCC of the oesophagus and this genotype correlated with poorer survival rates (Sturm *et al*, 2001). The M30 protein is a monoclonal antibody that recognises a neoepitope of cytokeratin 18 produced during apoptosis, thus serving as a marker of spontaneous apoptosis (Carr, 2000).

Allal *et al* (2003) assessed the prognostic significance of apoptotic regulatory proteins in anal carcinoma The proportion of tumours expressing Mcl-1 and Bcl-2 were 64 and 42%, respectively (Allal *et al*, 2003). Although Mcl-1 was found to have no prognostic significance, lack of Bcl-2 expression was an independent negative factor for local control (RR 4.66, *P*=0.0015) and also for DFS (RR 4.11, *P*=0.001) (Allal *et al*, 2003). In the same study, Bax and M30 expression was 42 and 12%, respectively, but neither showed correlation with clinical outcome in the 98 patients studied (Allal *et al*, 2003).

Two other studies have investigated Bcl-2 in patients with anal cancer, but there was no significant association with survival (Le *et al*, 2005; Ajani *et al*, 2009).

### Cyclins

Cyclins are a family of proteins that function in conjunction with CDKs to positively regulate specific cell cycle transition points (Bartek and Lukas, 2001). They have been studied extensively as regulators of cell cycle progression. The prognostic significance of three members of the cyclin family (A, D1 and E) has been studied in anal carcinoma (Table 5) (Allal *et al*, 2004; Le *et al*, 2005; Nilsson *et al*, 2006). All three cyclins govern cell cycle progression at the G1/S transition point, and cyclin A additionally exhibits regulatory control at the G2/M transition point (Pagano *et al*, 1992). Nilsson *et al* (2006) studied 215 patients with anal SCC and identified high cyclin A expression in 51% of these patients. This was significantly associated with better tumour-specific (81 *vs* 64%, *P*=0.009) and overall survival (77 *vs* 59%, *P*=0.005) when compared with tumours with low cyclin A expression (Nilsson *et al*, 2006). Furthermore, a lower rate of isolated locoregional failure was seen in patients with tumours expressing high levels of cyclin A (*P*<0.05) (Nilsson *et al*, 2006). Uni- and multi-variate analyses revealed that cyclin A was an independent predictive marker of survival (hazard ratio 0.54, 95% CI). In the two studies of cyclin D1 and one study of cyclin E in patients with anal cancer treated with radiotherapy±chemotherapy, pretreatment tumour levels of these proteins were found to have no association with patient outcome (Allal *et al*, 2004; Le *et al*, 2005).

### Markers of proliferation, invasion and metastasis

#### Ki-67 and MiB1

Ki-67 protein is a nuclear antigen expressed in proliferating cells. It can be detected within cells in all phases of the cell cycle except in G_0_ (Gerdes *et al*, 1983). This nuclear antigen expresses an epitope that is recognised by a murine monoclonal antibody known as MiB1, and therefore the number of Ki-67/MiB1-positive nuclei (termed the Ki-67/MiB1 index) can be used as a marker of cellular proliferation in paraffin-embedded tumour specimens.

Four studies have investigated the association between cellular proliferation in anal carcinoma as determined by the Ki-67/MiB1 index and clinical outcome (Table 6) (Allal *et al*, 1998; Grabenbauer *et al*, 1998; Indinnimeo *et al*, 2000b; Ajani *et al*, 2009). In the most recent of these studies by Ajani *et al* (2009), multivariate analysis identified Ki-67 as an independent predictor of DFS; a higher Ki-67 index correlated with longer DFS in patients treated with chemoradiotherapy (*P*=0.03). These findings are consistent with an earlier study identifying improved colostomy-free survival (90 *vs* 50%, *P*=0.04) in patients with tumours of higher proliferative potential as measured by the MiB1 index (Grabenbauer *et al*, 1998). Two others studies found no association between proliferative activity and patient survival in anal cancer (Allal *et al*, 1998; Indinnimeo *et al*, 2000b). One study did however report a significant correlation between Ki-67 index and depth of tumour invasion (*P*<0.05) and lymph node involvement (*P*<0.05) (Indinnimeo *et al*, 2000b).

These findings support a predictive role for Ki-67/MiB1 in the prognosis of anal cancer patients treated with chemoradiotherapy (Grabenbauer *et al*, 1998; Ajani *et al*, 2009). In these studies, high Ki-67/MiB1 positivity correlated with improved survival that may be related to heightened responses to antiproliferative chemotherapeutic agents. It is noteworthy that in the two studies where no prognostic significance was found, there were a cohort of patients who were not treated with chemotherapy but received radiotherapy (with or without local excision) (Allal *et al*, 1998; Indinnimeo *et al*, 2000b).

#### Proliferating cell nuclear antigen (PCNA)

PCNA is a nuclear protein associated with the late G_1_, S and early G_2_ phases of the cell cycle (Shin *et al*, 1994). It has a vital role in DNA synthesis and initiation of cellular proliferation. A single study has assessed the prognostic significance of PCNA in anal carcinoma, in which immunohistochemistry was used to analyse tumour samples obtained from 62 patients (Table 6) (Grabenbauer *et al*, 1998). There was no correlation with clinical outcome, and there have been no novel data identifying the prognostic value of PCNA in anal SCC.

#### Non-metastatic protein 23 (Nm23)

Studies of the nm23 protein both *in vitro* and *in vivo* have revealed a potential role in suppressing tumour metastasis through mechanisms that may be dependent on its nucleoside diphosphate kinase (NDPK) activity (MacDonald *et al*, 1995). Holm and Tanum (1996) studied nm23 expression using immunohistochemical analysis in 96 patients with anal carcinoma (Table 6). Of these specimens, 76 (79%) showed cytoplasmic nm23 staining, with 23 of these cases also exhibiting positive nuclear staining (Holm and Tanum, 1996). A correlation was identified between the presence of cytoplasmic nm23 and shorter overall survival (*P*=0.03), but no correlation was identified between nuclear nm23 staining and survival using univariate analysis (Holm and Tanum, 1996). These results are contradictory with respect to its proposed function as a suppressor of metastasis (Holm and Tanum, 1996). A smaller Italian study of the H1subtype of the nm23 protein in 22 anal cancer patients found no significant association between nm23 H1 expression and prognosis (Indinnimeo *et al*, 2000a).

#### MCM7

MCM7 belongs to a family of 6 minichromosome maintenance proteins (MCM 2–7) that have an essential role in DNA replication and can be detected throughout the cell cycle. They are degraded in cells that have exited the cell cycle, such as fully differentiated cells (Tachibana *et al*, 2005). They have documented potential as cancer biomarkers capable of predicting outcome in squamous neoplasms of the oral cavity (Kodani *et al*, 2003). Bruland *et al* (2008) studied MCM7 in anal carcinoma and found that high levels of the MCM7 gene product was significantly associated with improved relapse-free survival (*P*=0.017) and cancer-specific survival (*P*=0.011) using univariate analysis (Table 6).

#### Cathepsin D

Cathepsin D is a lysosomal hydrolase involved in proteolysis and is hypersecreted in neoplastic cells (Abbott *et al*, 2010). Excessive production of this protein in the tumour microenvironment promotes extracellular matrix degradation, thereby enhancing the invasive potential of cancerous cells (Abbott *et al*, 2010). Overexpression of Cathepsin D has been associated with poorer outcome in breast carcinoma (Niu *et al*, 2002; Brujan *et al*, 2009), although these findings have not consistently been replicated (Cocquyt *et al*, 2003; Anim *et al*, 2005).

A single study assessed the prognostic significance of Cathepsin D in anal cancer, and although 50% of tumours were Cathepsin D positive, the investigators found no correlation with overall survival (*P*>0.10) or with the clinicopathological parameters of tumour stage, tumour differentiation or lymph node involvement (Table 6) (Holm and Tanum, 1996).

### Angiogenesis

#### Vascular endothelial growth factor (VEGF)

Neovascularisation is key to tumour invasion and metastasis, and VEGF has an important role in this angiogenic process through interaction with its complimentary VEGF receptors (Backer *et al*, 2009). Anticancer therapies have been developed that target the VEGF receptor in order to improve tumour response rates. Two studies have investigated the prognostic significance of tumour VEGF levels in anal cancer patients treated with chemoradiotherapy, but neither study identified a correlation with patient survival (Table 7) (Wong *et al*, 1999; Ajani *et al*, 2009).

#### Microvessel density (MVD) and cluster of differentiation 31 (CD31)

In recent years, a widely established method of determining tumour vascularity has been through calculating MVD, which involves identifying vascular hot spots within the tumours and subsequently counting the number of individual microvessels seen (Pathak *et al*, 2008). MVD can thus be considered as a semiquantitative marker of angiogenesis. A number of endothelial surface markers have been used to examine MVD in malignant tumours, and among the three studies that have assessed the prognostic significance of MVD in anal cancer, one study used factor VIII (Wong *et al*, 1999) and two studies used the CD31 molecule (Table 7) (Indinnimeo *et al*, 2001; Nilsson *et al*, 2006). All three studies, however, failed to demonstrate a putative role for MVD in predicting anal cancer prognosis (Wong *et al*, 1999; Indinnimeo *et al*, 2001; Nilsson *et al*, 2006). The study by Indinnimeo *et al* (2001) did however report a positive correlation between CD31 score and depth of tumour invasion (*P*<0.05) in anal cancer, which supports the hypothesis that tumour growth occurs through angiogenesis-dependent mechanisms.

### Tumour markers

#### SCC antigen (SSCAg)

Serum concentrations of SCCAg, a polypeptide subunit of a protein originally identified in malignant human cervical tissue (Kato and Torigoe, 1977), is elevated in several tumour sites including the anal canal (Petrelli *et al*, 1988; Indinnimeo *et al*, 1997). Goldman *et al* (1993) examined the prognostic significance of this tumour marker and reported a clear association between SCCAg levels and survival (Table 8). Higher pretreatment serum SCCAg levels correlated with reduced tumour-free survival (*P*<0.00005) and overall survival (*P*=0.02) using multivariate analysis (Goldman *et al*, 1993). Another study of SCCAg in anal cancer found no prognostic value of pretreatment serum SCCAg, but did note that SCCAg levels were significantly elevated in patients who had relapsed following primary treatment (Fontana *et al*, 1991).

#### Carcinoembryonic antigen (CEA)

CEA, a complex glycoprotein that has long been established as a useful adjunct in monitoring patients with colorectal cancer (Goldstein and Mitchell, 2005), was investigated over a decade ago in patients with anal SCC undergoing combined chemoradiotherapy (Table 8) (Tanum *et al*, 1992). Of the patients, 19% were found to have raised serum CEA levels before treatment and 17.5% of tumours stained positive for CEA, but no correlation with clinical outcome was observed (Tanum *et al*, 1992).

### Sonic hedgehog (SHH) signalling

#### SHH and Gli-1

Sonic hedgehog is a secreted glycoprotein belonging to the hedgehog family of proteins that are essential to organogenesis (Ruiz i Altaba *et al*, 2002). SHH triggers a complex signal transduction pathway, the cellular response to which is mediated by three Gli proteins (Gli-1, Gli-2 and Gli-3) (Ruiz i Altaba *et al*, 2002). Gli-1 is a transcription factor that serves as an important regulator of mammalian hedgehog signalling (Ruiz i Altaba *et al*, 2002). Hedgehog signalling has been associated with failure to respond to chemoradiotherapy and also with increased incidence of tumour regrowth following treatment.

Ajani *et al* (2009) investigated the prognostic role in patients with anal cancer treated with chemoradiotherapy. Immunohistochemistry was used to measure tumour SHH and Gli-1 expression. Overexpression of both markers was demonstrated through multivariate analysis to be independent predictors of shorter DFS (*P*=0.02 and *P*=0.02, respectively; Table 8).

#### Human telomerase reverse transcriptase (hTERT)

Tumour proliferation and invasion is dependent on the activity of the telomerase enzyme that is responsible for extension of chromosomal ends (Deville *et al*, 2009). The hTERT protein forms a subunit of telomerase, and high levels of expression have been reported in several human malignancies including lung, bladder, oesophagus and colorectal tumours (de Kok *et al*, 2000). One study of hTERT in anal cancer failed to identify a relationship between expression of protein and patient outcome (Table 8) (Ajani *et al*, 2009).

## Discussion

Outcomes from the studies summarised in this review highlight a spectrum of biomarkers in anal cancer that may predict patient response to chemoradiation. Tumour-suppressor genes *p53* and *p21* have been shown to be of potential prognostic value in patients with anal cancer treated by chemoradiation, although these findings were not found to be universal. The discordant findings may at least in part, be explained by methodological differences such as the choice of detection antibodies used in the immunohistochemical analysis. The reliability of immunostaining in detection of p53 mutation remains uncertain, thereby affecting detection rates and subsequent clinical correlation (Neal *et al*, 2006). Mutational analysis using sequencing techniques has also been used for p53 detection in human cancers, but these techniques have not been employed in prognostic studies of anal carcinoma to date (Neal *et al*, 2006). Although clarification of the role of p53 and p21 is required in patients treated with chemoradiotherapy, further evaluation of their potential as novel therapeutic targets is also essential. Although not yet assessed in anal SCC, clinical trials of treatment of oesophageal SCC with adenovirus-mediated p53 gene transfer in combination with radiotherapy has been associated with improved local tumour control in unresectable tumours (Oohira *et al*, 2004). Simultaneous vector-mediated transduction of both p53 and p21 mRNA into hepatocellular and colorectal carcinoma cells performed in a recent study (Idogawa *et al*, 2009) was shown to increase rates of apoptotic cell death and also act synergistically with doxorubicin, thus having important therapeutic implications.

Since the establishment and effective use of trastuzumab (HER-2) in the treatment of breast carcinoma, there has been a drive to target the EGFR family of receptor proteins in other human cancers. None of these studies of EGFR in anal cancer have identified a direct link between EGFR status and prognosis after chemoradiation. However, the high levels of EGFR expression seen in anal tumours suggest that trials to investigate the activity of both TK inhibition and antibody inhibition of EGFR signalling are merited. It remains to be elucidated whether EGFR copy number (Hirsch *et al*, 2006) and EGFR mutation status (Zhu *et al*, 2008) will be markers of response to these agents in the context of anal SCC as has been shown for lung cancers. There are likely to be additional factors involved in determining patient responsiveness to these agents. The *K-RAS* gene, encoding a protein downstream of the EGFR receptor, has been associated with poorer responses to EGFR inhibitors when present in a mutated form. In the setting of colorectal cancer, it appears active only in colorectal cancers harbouring wild-type K-RAS (Amado *et al*, 2008). Recent data (Bonner *et al*, 2010) from a randomised trial of 424 patients with head and neck SCC revealed that survival was prolonged when anti-EGFR therapy (cetuximab) was combined with radiotherapy compared with radiotherapy alone. Clearly, other potential markers of response, such as B-RAF status, require assessment in this context. This also appears predominantly a ‘K-RAS wild-type’ disease according to a single small study (Zampino *et al*, 2009).

A single preliminary study suggested that NF-*κ*B may be of prognostic value in anal cancer, but there have not been any other confirmatory studies (Ajani *et al*, 2009). Studies of NF-*κ*B, most notably haematological malignancies, demonstrate that aberrant expression of this transcription factor is associated with tumour growth, progression and resistance to chemotherapeutic agents (Baud and Karin, 2009). Moreover, the use of the reversible 26S proteosome inhibitor bortezomib, which inhibits NF-*κ*B activity, is an effective and approved agent in the treatment of multiple myeloma (Baud and Karin, 2009). However, NF-*κ*B-targeted therapy is yet to become as widely established in other human malignancies.

Other apoptotic regulators that were shown to correlate with anal cancer prognosis in a single study were the Bcl-2 and M30 proteins (Allal *et al*, 2003). Two other studies of Bcl-2 failed to identify a correlation with clinical outcome (Le *et al*, 2005; Ajani *et al*, 2009). These studies had a relatively small patient sample size and therefore the true prognostic impact of the Bcl-2 and other apoptotic-regulatory molecules may need larger cohorts. In the study of apoptosis-regulating proteins by Allal *et al* (2003), subgroup analysis revealed that patients with Bcl-2-positive/p53-negative anal tumours had significantly higher 5-year DFS compared with patients with tumours expressing all other combinations of these two proteins. Furthermore, results from phase III trials of oblimerson (Genasense), an antisense nucleotide that targets Bcl-2, in chronic lymphocytic leukaemia showed improved survival rates only when given in conjunction with conventional chemotherapy but not when used alone (O’Brien *et al*, 2007). This suggests that future therapeutic targets may need to be used in conjunction with cytotoxic agents or other targeted treatments.

Data from two of the four studies of Ki-67/MiB1 supported a predictive role in the prognosis of anal cancer patients treated with chemoradiotherapy (Grabenbauer *et al*, 1998; Ajani *et al*, 2009). In these studies, high Ki-67/MiB1 positivity correlated with improved survival that may be related to heightened responses to antiproliferative chemotherapeutic agents. This is highlighted by the fact that in the two studies where no prognostic significance was found, there were a cohort of patients who were not treated with chemotherapy but received radiotherapy (with or without local excision) (Allal *et al*, 1998; Indinnimeo *et al*, 2000b). The question therefore remains as to whether the potential prognostic value of Ki-67/MiB1 is confined to certain treatment groups and if individuals with highly proliferative tumours as determined by their Ki-67/MiB1 index are likely to require more aggressive treatment regimens.

The oncological significance of the VEGF molecule is evident from the established use bevacizumab (Avastin), a humanised anti-VEGF monoclonal antibody in the treatment of metastatic breast, colorectal, renal and non-small cell lung carcinoma (Goerner *et al*, 2010). The two studies of VEGF in anal cancer reported no association with patient outcome (Wong *et al*, 1999; Ajani *et al*, 2009). The prognostic significance of VEGF in anal carcinoma needs further evaluation.

Investigation of molecular markers such as p16, PCNA, MVD and the Rb protein failed to demonstrate a prognostic utility in anal cancer. However, the degree to which these findings are limited by methodological factors is unclear. Other proteins such as M30, cyclin A, SCCAg, SHH and Gli-1 were demonstrated to be of predictive value in individual studies but further trials are required to reproduce these findings.

Other cancers related to HPV infection continue to increase in incidence. HPV-associated head and neck SCC is associated with HPV 16 similar to anogenital SCCs. *In situ* hybridisation studies have demonstrated that HPV status correlates (Kuo *et al*, 2008) with treatment response, progression-free survival and overall survival in oropharyngeal SCC (Fakhry *et al*, 2008; Ang *et al*, 2010). In this setting, much debate exists regarding the optimal detection technique and whether P-16 should be used as a surrogate marker. Future strategies may involve the application of such hypotheses to anal cancer with the potential for vaccine therapy in established HPV-associated disease. The National Cancer Institute Phase II trial has completed accrual in studying the effectiveness of human papillomavirus vaccine (HPV-16 E-6 and E-7 peptides) therapy in treating patients who have advanced or recurrent cancer of the cervix, vagina, penis, anus, oesophagus, or head and neck (National Cancer Institute, 2009). We await the results of these and other studies.

There is a growing appreciation of the fact that all clinical trials of emerging targeted treatments must incorporate biomarkers studies in order to minimise the possibility of failing to appreciate significant activity of the drug in a biological subset of a given cancer type and to maximise the chance of response in a given patient. This is all the more essential in the case of anal carcinoma, which is an uncommon cancer type that will limit the patient numbers available for clinical trials. Certainly, there are targeted treatments that have been shown to have activity in other EGFR-expressing cancers that need assessment in this tumour type, and biomarkers that have been shown to be helpful in other cancer sites should be prioritised for assessment. Any biomarker studies and validation thereof should aim to comply with Biomarker Task Force recommendations in order to ensure that results can be assessed in terms of realistic clinical utility (Dancey *et al*, 2010).

## Conclusion

Biomarkers in anal cancer may possess the ability to tailor treatment to individuals. Certain markers such as the EGFR protein are potential treatment targets in this disease. It remains to be seen if biomarkers of response to anti-EGFR therapies shown to be of use in other carcinoma types are applicable to anal carcinoma. Larger prospective studies of single or panels of multiple biomarkers are necessary with standardised methodology. Novel study designs are required and future prospects include the use of DNA microarray-based gene expression profiling that has already been shown to provide valuable prognostic information in patients with breast cancer (Munkacsy *et al*, 2010). Pharmacogenomics has the potential to make a major contribution to biomarker identification and future directions of therapy in anal cancer. Within its realm, single-nucleotide polymorphism (SNP) analysis serves as a promising avenue for future research studies. Although the quest for prognostic markers in anal cancer is currently in its early stages, expanding research into this field is driven by the prospect of unravelling molecular characteristics of these tumours that may ultimately prolong patient survival and reduce the health and economic burden of this disease epidemic.

## Figures and Tables

**Table 1:** Studies of tumour-suppressor genes (*p53*) in anal carcinoma

**Authors**	**Year**	**Biomarker**	**Patients (*n*)** a	**Method**	**Radio±chemotherapy (*n*)**	**Primary surgical treatment (*n*)**	**Mean (median) follow-up period in months**	**Biomarker +ve patients** b	**Survival (biomarker +ve *vs* −ve patients)**	**Correlation with prognosis**
Tanum and Holm	1996	p53	97	IHC	97 chemoradiotherapy	0	60	34%	Not reported	No prognostic significance identified
Bonin *et al*	1999	p53	64	IHC	64 chemoradiotherapy	0	(57)	48%	48 *vs* 33% DFS	No prognostic significance identified
Indinnimeo *et al*	1999	p53	14	IHC	Not reported	Not reported	57.6	60%	44 *vs* 40% DFS	No prognostic significance identified
Wong *et al*	1999	p53	49	IHC	49 chemoradiotherapy	0	(54)	51%	62 *vs* 67% 5-year DFS	p53 expression associated with reduced DFS (*P*=0.01)
Allal *et al*	2003	p53	98	IHC	47 radiotherapy alone 51 chemoradiotherapy	0	(124)	43%	75 *vs* 55% 5-year DFS	p53 expression associated with reduced local tumour control (*P*=0.03) and reduced DFS (0.003)
Le *et al*	2005	p53	21	IHC	21 chemoradiotherapy	0	Not reported	100%	Not reported	No prognostic significance identified
Nilsson *et al*	2006	p53	214	IHC	Not reported	7 local excision, 6 APR	Not reported	50%	Not reported	No prognostic significance identified
Ajani *et al*	2009	p53	30	IHC	30 chemoradiotherapy	0	Not reported	Not reported	RR=1.22 for DFS	No prognostic significance identified

Abbreviations: APR=abdominoperineal resection; DFS=disease-free survival; IHC=immunohistochemistry; Rb=retinoblastoma; RR=relative risk.

a Patients included in final biomarker analysis.

b In studies where patients have been differentiated by the presence or absence of tumour biomarker overexpression, overexpression is considered as biomarker positive.

**Table 2:** Studies of tumour suppressor-genes in anal carcinoma

**Authors**	**Year**	**Biomarker**	**Patients (*n*)** a	**Method**	**Radio±chemotherapy (*n*)**	**Primary surgical treatment (*n*)**	**Mean (median) follow-up period in months**	**Biomarker +ve patients** b	**Survival (biomarker +ve *vs* −ve patients)**	**Correlation with prognosis**
Holm *et al*	2001	p21	94	IHC	94 chemoradiotherapy	0	60	71%	Not reported	Lack of p21 expression associated with reduced overall survival (*P*=0.013)
Allal *et al*	2004	p21	98	IHC	47 radiotherapy alone 51 chemoradiotherapy	0	(124)	65%	66 *vs* 59% 5-year DFS	No prognostic significance identified
Nilsson *et al*	2006	p21	215	IHC	Not reported	7 local excision, 6 APR	Not reported	69%	71 *vs* 62% 5 year overall survival	Absence of p21 associated with an increased locoregional failure rate (*P*<0.05)
Holm *et al*	2001	p27	94	IHC	94 chemoradiotherapy	0	60	75%	Not reported	No prognostic significance identified
Bruland *et al*	2008	p16	55	IHC	9 radiotherapy alone 46 chemoradiotherapy	0	86.4	Not reported	Not reported	No prognostic significance identified
Ajani *et al*	2009	p16	30	IHC	30 chemoradiotherapy	0	Not reported	Not reported	RR=0.81 for DFS	No prognostic significance identified
Tanum and Holm	1996	Rb	97	IHC	97 chemoradiotherapy	0	(60)	95%	Not reported	No prognostic significance identified

Abbreviations: APR=abdominoperineal resection; DFS=disease-free survival; IHC=immunohistochemistry; Rb=retinoblastoma; RR=relative risk.

a Patients included in final biomarker analysis.

b In studies where patients have been differentiated by the presence or absence of tumour biomarker overexpression, overexpression is considered as biomarker positive.

**Table 3:** Studies of the EGFR family in anal carcinoma

**Authors**	**Year**	**Biomarker**	**Patients (*n*)** a	**Method**	**Radio±chemotherapy (*n*)**	**Primary surgical treatment (*n*)**	**Mean (median) follow-up period in months**	**Biomarker +ve patients** b	**Survival (biomarker +ve *vs* −ve patients)**	**Correlation with prognosis**
Tanum and Holm	1996	c-erb B-2/HER-2	97	IHC	97 chemoradiotherapy	0	60	0	Not reported	No prognostic significance identified
Le *et al*	2005	HER-2	21	IHC	21 chemoradiotherapy	0	Not reported	0	Not reported	No prognostic significance identified
Le *et al*	2005	EGFR/HER-1	21	IHC	21 chemoradiotherapy	0	Not reported	100%	Not reported	High EGFR expression seen in all 21 specimens
Alvarez *et al*	2006	EGFR/HER-1	38	IHC and FISH	38 chemoradiotherapy	0	22.6	55%	Not reported	No prognostic significance identified
Ajani *et al*	2009	EGFR/HER-1	30	IHC	30 chemoradiotherapy	0	Not reported	87%	RR=0.40 for DFS	No prognostic significance identified

Abbreviations: EGFR=epidermal growth factor receptor; IHC=immunohistochemistry; FISH=fluorescent *in situ* hybridisation; RR=relative risk.

a Patients included in final biomarker analysis.

b In studies where patients have been differentiated by the presence or absence of tumour biomarker overexpression, overexpression is considered as biomarker positive.

**Table 4:** Studies of apoptotic regulators in anal carcinoma

**Authors**	**Year**	**Biomarker**	**Patients (*n*)** a	**Method**	**Radio±chemotherapy (*n*)**	**Primary surgical treatment (*n*)**	**Mean (median) follow-up period in months**	**Biomarker +ve patients** b	**Survival (biomarker +ve *vs* −ve patients)**	**Correlation with prognosis**
Ajani *et al*	2009	NF-*κ*B	30	IHC	30 chemoradiotherapy	0	Not reported	Not reported	RR=1.05 for DFS	Higher levels of NF-*κ*B associated with shorter DFS (*P*=0.002)
Allal *et al*	2003	Bax	98	IHC	47 radiotherapy alone 51 chemoradiotherapy	0	(124)	29%	61 *vs* 65% 5-year DFS	No prognostic significance identified
Allal *et al*	2003	Bcl-2	98	IHC	47 radiotherapy alone 51 chemoradiotherapy	0	(124)	58%	56 *vs* 75% 5-year DFS	Bcl-2 expression associated with improved local tumour control (*P*=0.0015) and DFS (*P*=0.001)
Le *et al*	2005	Bcl-2	21	IHC	21 chemoradiotherapy	0	Not reported	24%	Not reported	No prognostic significance identified
Ajani *et al*	2009	Bcl-2	30	IHC	30 chemoradiotherapy	0	Not reported	Not reported	RR=0.90 for DFS	No prognostic significance identified
Allal *et al*	2003	Mcl-1	98	IHC	47 radiotherapy alone 51 chemoradiotherapy	0	(124)	36%	60 *vs* 66% 5-year DFS	No prognostic significance identified
Allal *et al*	2003	M30	98	IHC	47 radiotherapy alone 51 chemoradiotherapy	0	(124)	88%	67 *vs* 37% 5-year DFS	Presence of M30 associated with lower local control (*P*=0.034) and lower DFS (*P*=0.03)

Abbreviations: DFS=disease-free survival; IHC=immunohistochemistry; NF-*κ*B=nuclear factor-*κ*B; RR=relative risk.

a Patients included in final biomarker analysis.

b In studies where patients have been differentiated by the presence or absence of tumour biomarker overexpression, overexpression is considered as biomarker positive.

**Table 5:** Studies of cyclins in anal carcinoma

**Authors**	**Year**	**Biomarker**	**Patients (*n*)** a	**Method**	**Radio±chemotherapy (*n*)**	**Primary surgical treatment (*n*)**	**Mean (median) follow-up period in months**	**Biomarker +ve patients** b	**Survival (biomarker +ve *vs* −ve patients)**	**Correlation with prognosis**
Nilsson *et al*	2006	Cyclin A	215	IHC	Not reported	7 local excision, 6 APR	Not reported	51%	77 *vs* 59% 5-year overall survival	High cyclin A expression associated with improved overall (*P*=0.005) and DFS(*P*=0.009)
Allal *et al*	2004	Cyclin D1	98	IHC	47 radiotherapy alone 51 chemoradiotherapy	0	(124)	34%	57 *vs* 67% 5-year DFS	No prognostic significance identified
Le *et al*	2005	Cyclin D1	21	IHC	21 chemoradiotherapy	0	Not reported	33%	Not reported	No prognostic significance identified
Allal *et al*	2004	Cyclin E	98	IHC	47 radiotherapy alone 51 chemoradiotherapy	0	(124)	51%	61 *vs* 67% 5-year DFS	No prognostic significance identified

Abbreviations: APR=abdominoperineal resection; DFS=disease-free survival; IHC=immunohistochemistry.

a Patients included in final biomarker analysis.

b In studies where patients have been differentiated by the presence or absence of tumour biomarker overexpression, overexpression is considered as biomarker positive.

**Table 6:** Studies of markers of proliferation, invasion and metastasis in anal carcinoma

**Authors**	**Year**	**Biomarker**	**Patients (*n*)** a	**Method**	**Radio±chemotherapy (*n*)**	**Primary surgical treatment (*n*)**	**Mean (median) follow-up period in months**	**Biomarker +ve patients** b	**Survival (biomarker +ve *vs* −ve patients)**	**Correlation with prognosis**
Allal *et al*	1998	MiB1	55	IHC	31 chemoradiotherapy 14 radiotherapy only	0	(94)	41%	59 *vs* 67% 5-year DFS	No prognostic significance identified
Grabenbauer *et al*	1998	MiB1	62	IHC	62 chemoradiotherapy	0	(52)	34%	73 *vs* 50% 5-year DFS	Higher MiB1 index associated with better colostomy-free survival (*P*=0.04)
Indinnimeo *et al*	2000a,b	Ki-67	31	IHC	13 chemoradiotherapy 7 radiotherapy followed by local excision	5 local excision, 6 APR	74.4	65%	Not reported	No prognostic significance identified
Ajani *et al*	2009	Ki-67	30	IHC	30 chemoradiotherapy	0	Not reported	Not reported	RR=0.98 for DFS	Higher levels of Ki-67 associated with longer DFS (*P*=0.005)
Grabenbauer *et al*	1998	PCNA	62	IHC	62 chemoradiotherapy	0	(52)	32%	78 *vs* 45% 5-year DFS	No prognostic significance identified
Holm and Tanum	1996	nm23	96	IHC	96 chemoradiotherapy	0	60	79%	Not reported	Cytoplasmic nm23 expression associated with shorter survival *P*=0.03)
Indinnimeo *et al*	2000a,b	nm23	22	IHC	9 chemoradiotherapy 5 chemotherapy	2 local excision 6 APR	63.6	27%	Not reported	No prognostic significance identified
Bruland *et al*	2008	MCM7	55	IHC	9 radiotherapy alone 46 chemoradiotherapy	0	86.4	Not reported	Not reported	High MCM7 correlated with improved cancer-specific survival (*P*=0.011)
Holm and Tanum	1996	Cathepsin D	96	IHC	96 chemoradiotherapy	0	60	50%	Not reported	No prognostic significance identified

Abbreviations: APR=abdominoperineal resection; DFS=disease-free survival; IHC=immunohistochemistry; PCNA=proliferating cell nuclear antigen; MCM7=mini-chromosome maintenance protein 7; nm23=non-metastatic protein 23; RR=relative risk.

a Patients included in final biomarker analysis.

b In studies where patients have been differentiated by the presence or absence of tumour biomarker overexpression, overexpression is considered as biomarker positive.

**Table 7:** Studies of markers of angiogenesis in anal carcinoma

**Authors**	**Year**	**Biomarker**	**Patients (*n*)** a	**Method**	**Radio±chemotherapy (*n*)**	**Primary surgical treatment (*n*)**	**Mean (median) follow-up period in months**	**Biomarker +ve patients** b	**Survival (biomarker +ve *vs* −ve patients)**	**Correlation with prognosis**
Wong *et al*	1999	VEGF	36	IHC	36 chemoradiotherapy	0	(54)	100% (no cutoff level of expression reported)	Not reported	No prognostic significance identified
Ajani *et al*	2009	VEGF	30	IHC	30 chemoradiotherapy	0	Not reported	Not reported	RR=0.98 for DFS	No prognostic significance identified
Wong *et al*	1999	MVD	35	IHC	35 chemoradiotherapy	0	(54)	51%	78 *vs* 87% 5-year DFS	No prognostic significance identified
Indinnimeo *et al*	2001	CD31 (MVD)	24	IHC	16 chemoradiotherapy	2 local excision, 6 APR	(75.6)	50%	Not reported	No prognostic significance identified
Nilsson *et al*	2006	CD31 (MVD)	209	IHC	Not reported	7 local excision, 6 APR	Not reported	50%	Not reported	No prognostic significance identified

Abbreviations: APR=abdominoperineal resection; DFS=disease-free survival; IHC=immunohistochemistry; VEGF=vascular endothelial growth factor; MVD=microvessel density; CD31=cluster of differentiation 31; RR=relative risk.

a Patients included in final biomarker analysis.

b In studies where patients have been differentiated by the presence or absence of tumour biomarker overexpression, overexpression is considered as biomarker positive.

**Table 8:** Studies of tumour markers, hedgehog signalling and telomerase activity in anal carcinoma

**Authors**	**Year**	**Biomarker**	**Patients (*n*)** a	**Method**	**Radio±chemotherapy (*n*)**	**Primary surgical treatment (*n*)**	**Mean (median) follow-up period in months**	**Biomarker +ve patients** b	**Survival (biomarker +ve *vs* −ve patients)**	**Correlation with prognosis**
Fontana *et al*	1991	SCCAg	66	RIA	Not reported	Not reported	42.4	54%	Not reported	Correlation with relapse only in post-treatment samples
Goldman *et al*	1993	SCCAg	60	RIA	59 radiotherapy	1 APR	(42)	33%	43 *vs* 81% 5-year overall survival	Elevated SCCAg level correlated with reduced overall survival (*P*=0.02) and tumour-free survival (*P*<0.00005).
Tanum *et al*	1992	CEA	106	RIA and tissue staining	106 chemoradiotherapy	0	65.2	19%	Not reported	No prognostic significance identified
Ajani *et al*	2009	SHH	30	IHC	30 chemoradiotherapy	0	Not reported	Not reported	RR=1.03 for DFS	Higher levels of SHH associated with shorter DFS (*P*=0.02)
Ajani *et al*	2009	Gli-1	30	IHC	30 chemoradiotherapy	0	Not reported	Not reported	RR=1.03 for DFS	Higher levels of Gli-1 associated with shorter DFS (*P*=0.02)
Ajani *et al*	2009	hTERT	30	IHC	30 chemoradiotherapy	0	Not reported	Not reported	RR=0.98 for DFS	No prognostic significance identified

Abbreviations: APR=abdominoperineal resection; DFS=disease-free survival; RAI=radioimmunoassay; SCCAg=squamous cell carcinoma antigen; CEA=carcinoembryonic antigen; SHH=sonic hedgehog; hTERT=human telomerase reverse transcriptase; RR=relative risk.

a Patients included in final biomarker analysis.

b In studies where patients have been differentiated by the presence or absence of tumour biomarker overexpression, overexpression is considered as biomarker positive.
